# Noninvasive papillary urothelial carcinoma with pathological features in between low and high grades: A case report

**DOI:** 10.1097/MD.0000000000025693

**Published:** 2021-04-30

**Authors:** Shuang Ma, Yang Han, Di Zhang, Qingchang Li, Enhua Wang, Guangping Wu, Huanyu Zhao

**Affiliations:** a Department of Neurology, Sheng Jing Hospital of China Medical University; b Department of Pathology, The First Affiliated Hospital and College of Basic Medical Sciences, China Medical University, Shenyang, Liaoning, China.

**Keywords:** case report, ki67, noninvasive papillary urothelial carcinoma, p53

## Abstract

**Rationale:**

Urinary bladder urothelial carcinoma is the most common malignant tumor in the urinary system, and noninvasive papillary urothelial carcinoma (NIPUC) comprises most bladder malignancies. NIPUC grading is important for therapeutic and clinical protocol selection. Here, we report a case of NIPUC with pathological features in between low (LG-NIPUC) and high (HG-NIPUC) grades NIPUC.

**Patient concerns:**

A 72-year-old male, presenting with a 20-year history of hypertension and 5 months of hematuria.

**Diagnoses:**

Computed tomography examination revealed a tumor in the urinary bladder neck. Microscopic investigation revealed that most tumor tissue samples had a branching papillary architecture, with well-developed fibrous-vascular cores. Tumor cells were slightly crowded, with somewhat altered cell polarity and cell adhesion. Immunohistochemistry showed positive Ki67 staining, mostly in the basal layer, while p53 staining was rarely positive. These samples were diagnosed as LG-NIPUC. However, a few tumor tissue samples presented mildly fused papillary architectures without cell polarity or adhesion. Most nuclei stained intensely and were pleomorphic. All epithelial tissue layers were ki67 positive, and the p53 positive rate was higher than that in the LG samples. Therefore, these were classified as HG-NIPUC.

**Interventions:**

The tumor was completely resected during lithotomy postural surgery.

**Outcomes:**

The patient is alive with a good recovery during 3 months after the surgery.

**Lessons:**

We diagnosed this patient as having LG-NIPUC with local HG-NIPUC components. HG- and LG-HIPUC have different outcomes. This case is a new challenge for the pathological grading of NIPUC. An intermediate HIPUC grade might need to be added to the original NIPUC grading system.

## 1 Introduction

Urinary bladder urothelial carcinoma is the most common malignant tumor in the urinary system, and noninvasive urothelial neoplasms represent the majority of bladder malignancies at initial diagnosis.^[1]^ Noninvasive urothelial neoplasms were introduced into the World Health Organization (WHO) classification system of urinary system tumors in 1973 and were then retained and updated in the WHO 2004 and 2016 classification system.^[2–4]^ The WHO classification summarized the morphology of urothelial neoplasms and presented divergent differentiation and genomic landscapes. Knowledge of the tumor morphology and genotype is important for therapeutic and clinical protocol selection for patients with urothelial neoplasms. Pathological grading of urothelial tumors is of particular importance in noninvasive urothelial neoplasms, specifically papillary neoplasms. Noninvasive papillary urothelial carcinoma (NIPUC) can be divided into low grade (LG) and high grade (HG), which differ in their pathological features and clinical prognosis.^[3,4]^ Here, we report a case of NIPUC with pathological features that lies in between LG and HG.

## 2 Case presentation

A 72-year-old Chinese male was diagnosed with hypertension in 2000 and received amlodipine (Norvasc Amlodipine Besylate, 5 mg/day) for many years. The patient was first admitted to the First Affiliated Hospital of China Medical University in September 2020. Before hospitalization, the cardinal manifestation of the patient was gross hematuria for over 5 months, and it was getting worse. The patient's diet, defecation, and weight were normal. There was no tenderness in the bladder area.

Hospital examination showed consistent bilateral thoracic respiratory movement; voiceless lung percussion, with no dry and wet rales; no liver or kidney pain and no shifting dullness on percussion; normal bowel sounds with no splashing sound during auscultation; and no tenderness or pain on tapping in the vertebral region. The patient was negative for the Babinski sign, ankle clonus, Hoffmann sign, Kernig sign, Brudzinski sign, and asterixis.

The patient had slightly high total-prostate-specific antigen (4.060 ng/mL; normal range, 0.000–4.000 ng/mL).

Computed tomography scans demonstrated a 1.2 × 1.1 cm filling defect on the posterior wall of the bladder, with obscured margins. Computed tomography attenuation values were 39 Hounsfield units in normal scanned images and 66 Hounsfield units after enhanced scanning. No abnormal retroperitoneal lymph nodes were observed.

**Figure 1:**
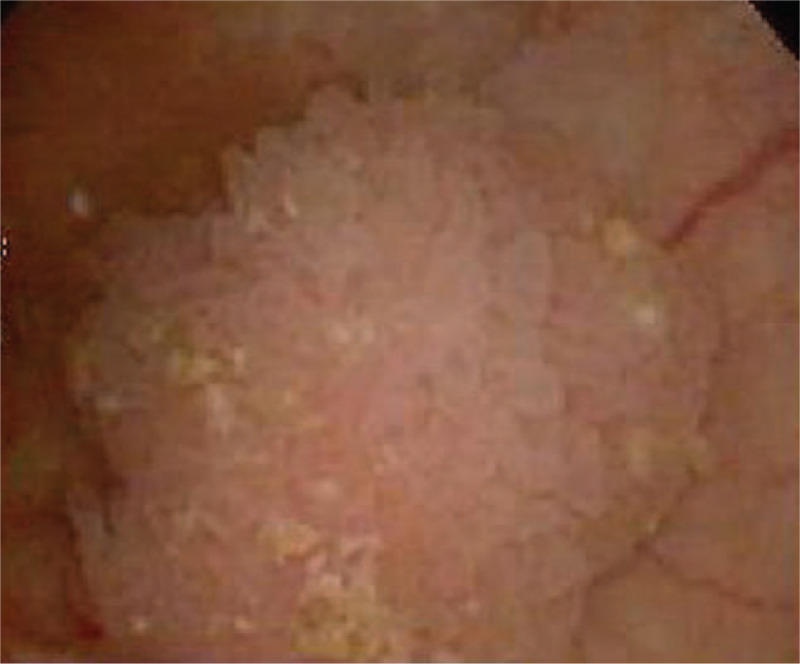
Flexible cystoscopy showed a cauliflower-like tumor.

Flexible cystoscopy showed a cauliflower-like tumor at the 8 o’clock position on the bladder neck, measuring 1.2 × 1.0 cm (Fig. 1). The patient underwent lithotomy postural surgery under epidural anesthesia. The tumor was completely resected to the deep muscularis by an electric needle knife.

**Figure 2:**
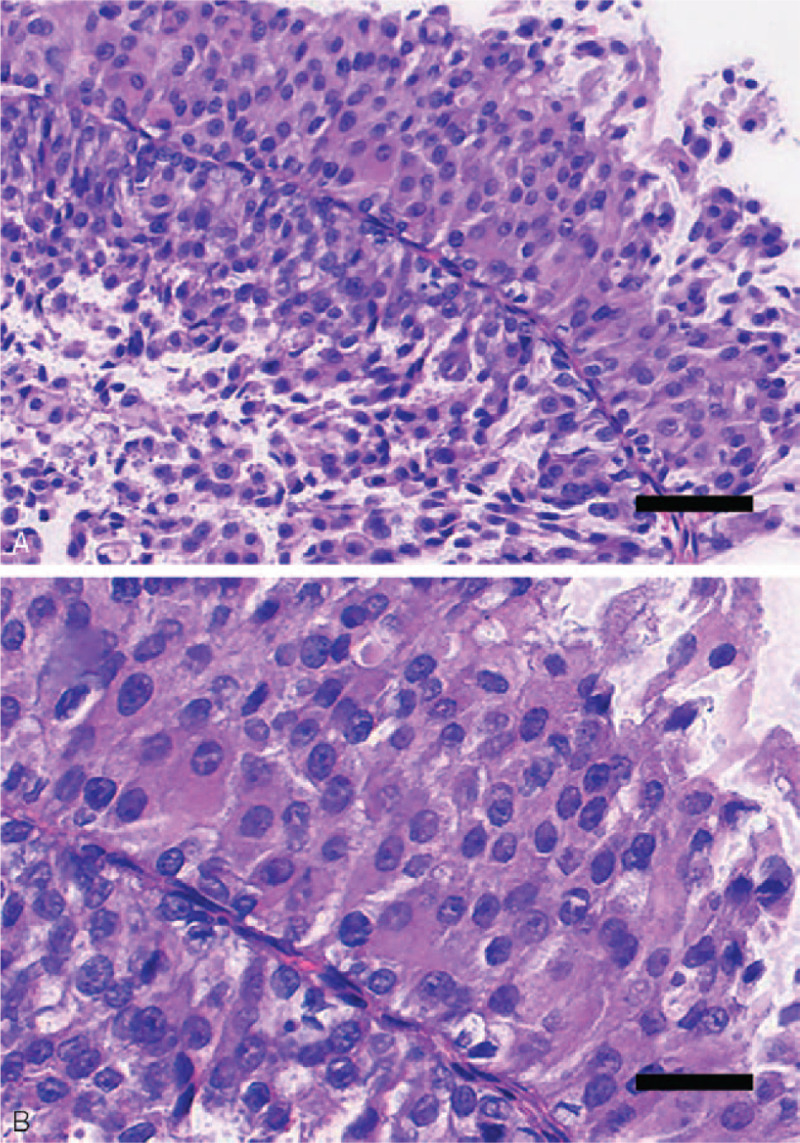
Histological features (hematoxylin and eosin staining) of low-grade noninvasive papillary urothelial carcinoma (LG-NIPUC). Scale bars: 1 mm (A), 400 μm (B).

**Figure 3:**
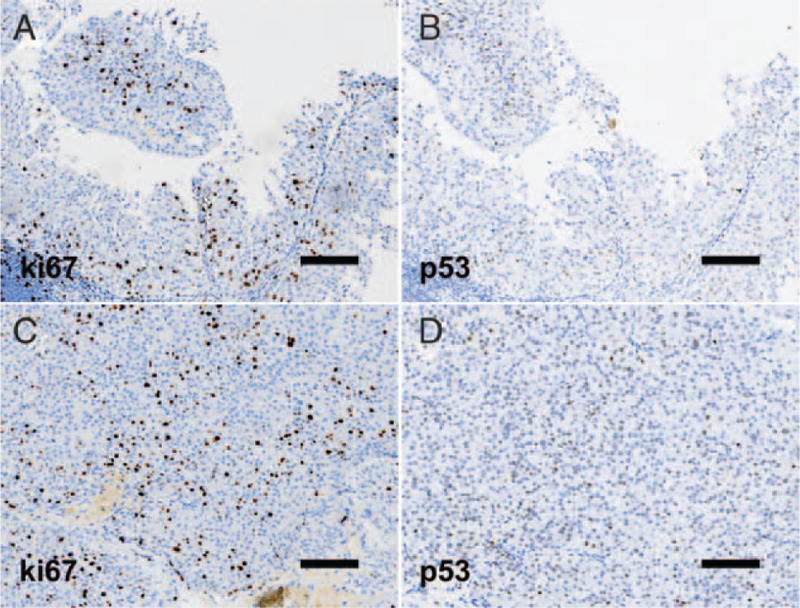
Immunohistochemistry examination in low-grade noninvasive papillary urothelial carcinoma (LG-NIPUC) (A, B) and high-grade noninvasive papillary urothelial carcinoma (HG-NIPUC) (C, D). Scale bars: 3 mm (A-D).

Microscopically, most tumor tissue samples were characterized by exophytic tumor growth with branching papillary architectures and well-developed branching fibrous-vascular papillary cores. Cells were somewhat crowded, with slightly altered polarity and cell adhesion (Fig. 2A). Cellular atypia was not evident, as the cells exhibited a nucleo-cytoplasmic ratio of about 50%. The tumor cells consisted of lightly eosinophilic cytoplasm and small distinct nucleoli. Nuclear atypia was mild, and the amount of karyokinesis per 10 high-power fields was small (Fig. 2B). Immunohistochemistry showed that ki67 positive staining was mostly located in the basal layer, while p53 staining was rarely positive (Fig. 3A, B). Such sections were diagnosed as LG-HIPUC.

**Figure 4:**
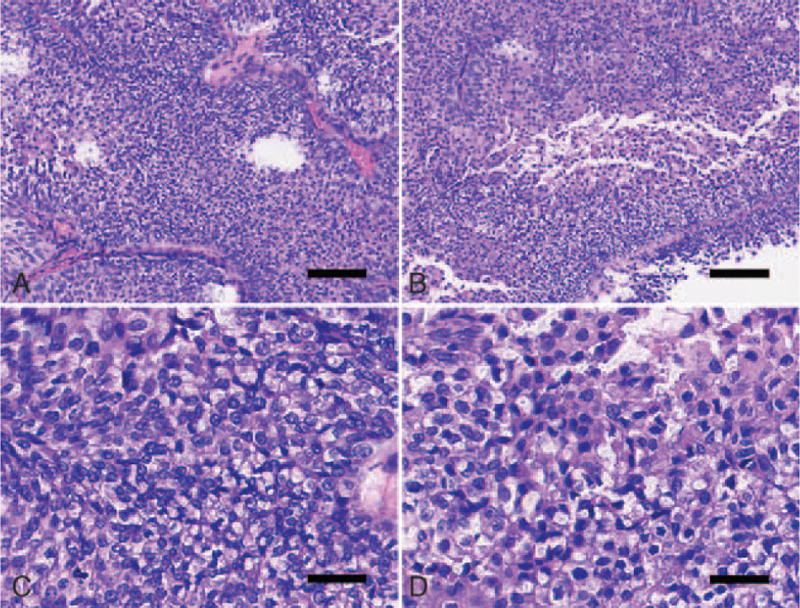
Histological features (hematoxylin and eosin staining) of high-grade noninvasive papillary urothelial carcinoma (HG-NIPUC). Scale bars: 3 mm (A, B), 400 μm (C, D).

A small number of tumor tissue samples comprised mildly fused papillary architectures (Fig. 4A, B). Cellular atypia was evident, exhibiting a nucleo-cytoplasmic ratio of about 90%. The cells were disorderly arranged, with no cell polarity or adhesion. Most nuclei were prominent, deeply stained, and pleomorphic. Mitotic count per 10 high-power fields was 5 to 10 (Fig. 4C, D). Immunohistochemical staining for ki67 was positive in all epithelial layers, and its positive rate was higher than that in LG areas. The p53 positive rate was higher than that in LG areas (Fig. 3C, D). Therefore, these samples were diagnosed as HG-HIPUC. Based on these findings, the patient was diagnosed as having LG-NIPUC with local HG-NIPUC components. Until now this patient was alive with no tumor recurrence (6 months).

## 3 Methodology

We fixed the tissues with 10% formalin and embedded them in paraffin. Paraffin block was cut into sections (4 μm) and then H&E staining and immunohistochemistry staining were used. A strep-tavidin peroxidase system (Ultrasensitive; Mai Xin, Fuzhou, China) was performed for immunohistochemistry staining. The primary antibodies used for immunohistochemistry staining were as following: p53, and Ki67 (Mai Xin, Fuzhou, China). The sections were examined under a microscope (OLYMPUS). The ethics committee of the First Affiliated Hospital of China Medical University approved this study.

## 4 Discussion and conclusions

Most bladder urothelial tumors are noninvasive at diagnosis (stage Ta).^[5,6]^ Pathological grade determination is important for predicting the outcome. LG-NIPUC shows frequent recurrence, rarely progresses to high grade, and has a good prognosis. HG-NIPUC shows frequent recurrence, stromal invasion and aggressive progression, and has a poor prognosis. Therefore, accurate pathological diagnosis is essential for bladder cancer patients.

According to the WHO classification, LG-NIPUC comprises orderly arranged papillary leaves, thin multibranched and slightly fused papillae, and branching fibrous-vascular core. Nuclear polarity and relatively consistent morphology can be distinguished. The nucleus shape and chromatin distribution might be slightly changed. The nucleolus is not apparent, and mitosis is rare. It is easy to distinguish its histological and cytological manifestations, even under low-power microscopy.

HG-NIPUC is composed of disordered papillary structures with moderate to prominent cellular atypia. This tumor grade is characterized by noninvasive papillary structures. Some papillae are thin, with frequent fusion and branching formation. The nuclei are pleomorphic, with moderate to significant changes in size, irregular chromatin distribution, prominent nucleoli, and frequent pathological mitosis, which can be seen through the entire epithelial layer (including the surface layer). The urothelium is locally thickened, varying degrees of atypical epithelial cell fusion are observed, and cell adhesion decreases.

In the present case, most tumor tissue samples complied with LG-NIPUC histopathological diagnostic standards, but small parts of the tumor tissue had fused papillary architectures and cellular atypia, following the definition of HG-NIPUC.

Since the WHO introduced the bladder tumors grading classification, several studies have focused on grading based on immunohistochemical markers.^[7,8]^ The *TP53* gene is a tumor suppressor gene involved in cellular damage response and cell-cycle arrest.^[9]^ This gene is located on 17p13.1. The protein product of *TP53*, p53, was shown to be mutated in many cancers, where inactive mutant forms may easily be detected. In bladder cancer, *TP53* mutations mostly occur in poorly differentiated tumors. The mutation rate can predict the recurrence and progression of bladder cancer and the death of these patients. The positive expression rate of p53 was shown to differ between tumor grades,^[10]^ and the recurrence rate was higher when p53 was highly expressed. According to previous studies, staining was considered positive when over 10% of the nucleus stained for p53.^[11]^

ki67 is a nuclear protein complex and a marker for cellular proliferation. The ki67 labeling index is an independent predictor of tumor proliferation, which is important for the WHO grading system. It is used to measure tumor proliferation in noninvasive bladder urothelial cell carcinoma and was shown to correlate with tumor recurrence, stage progression, and overall survival.^[12]^ Thus, ki67 LI could help stratify patients with noninvasive bladder tumors into risk level categories. Therefore, immunohistochemical detection of p53 and ki67 was used to predict bladder cancer progression.^[13]^

The basal layer, also known as the germinal layer, produces new cells to replace the aging and exfoliated keratinocytes, so the mitosis rate is high in the basal layer. The positive expression of ki67 in LG-NIPUC is primarily in the basal layer, and p53-positive staining is rare; however, both stain positive in HG-NIPUC, covering larger areas than in LG.

In summary, we diagnosed this patient as having LG-NIPUC with local HG components. HG- and LG-HIPUC have different outcomes. However, our detection methods are limited and need to be further improved. Our findings present a new challenge for pathological grading of NIPUC. We suggest that an intermediate HIPUC grade be added to the standard grading system.

## Author contributions

**Conceptualization:** Huanyu Zhao.

**Data curation:** Qingchang Li, Huanyu Zhao.

**Formal analysis:** Shuang Ma, Qingchang Li, Enhua Wang, Huanyu Zhao.

**Funding acquisition:** Huanyu Zhao.

**Investigation:** Yang Han, Di Zhang, Guangping Wu.

**Methodology:** Di Zhang, Huanyu Zhao.

**Supervision:** Enhua Wang, Guangping Wu, Huanyu Zhao.

**Validation:** Yang Han, Di Zhang, Huanyu Zhao.

**Visualization:** Shuang Ma, Huanyu Zhao.

**Writing – original draft:** Shuang Ma, Huanyu Zhao.

**Writing – review & editing:** Shuang Ma, Huanyu Zhao.

## References

[1] Siegel RL Miller KD Jemal A. Cancer statistics, 2015. CA Cancer J Clin 2015;65:05–29. 10.3322/caac.21254 25559415

[2] Mostofi FK Sobin LH Torloni H. Histological Typing of Urinary Bladder Tumours. Geneva: World Health Organization; 1973.

[3] Eble JN Sauter G Epstein JI. World Health Organization Classification of Tumours of the Urinary System and Male Genital Organs. Lyon: IARC Press; 2004.

[4] Moch H Humphrey PA Ulbright TM. World Health Organization Classification of Tumours of the Urinary System and Male Genital Organs. Lyon: International Agency for Research on Cancer; 2016.

[5] Ottley EC Pell R Brazier B. Greater utility of molecular subtype rather than epithelial-to-mesenchymal transition (EMT) markers for prognosis in high-risk non-muscle-invasive (HGT1) bladder cancer. J Pathol Clin Res 2020;6:238–51. 32374509 10.1002/cjp2.167 PMC7578305

[6] Lopez-Beltran A Montironi R. Non-invasive urothelial neoplasms: according to the most recent WHO classification. Eur Urol 2004;46:170–6. 15245809 10.1016/j.eururo.2004.03.017

[7] Rodriguez Pena MDC Chaux A Eich ML. Immunohistochemical assessment of basal and luminal markers in non-muscle invasive urothelial carcinoma of bladder. Virchows Arch 2019;475:349–56. 31300876 10.1007/s00428-019-02618-5

[8] Makboul R Hassan HM Refaiy A. A simple immunohistochemical panel could predict and correlate to clinicopathologic and molecular subgroups of urinary bladder urothelial carcinoma. Clin Genitourin Cancer 2019;17:e712–9. 31085058 10.1016/j.clgc.2019.04.011

[9] Jentsch M Snyder P Sheng C. p53 dynamics in single cells are temperature-sensitive [sci rep:1481]. Sci Rep 2020;10:1481. 32001771 10.1038/s41598-020-58267-1 PMC6992775

[10] Liu A Xue Y Liu F. Prognostic value of the combined expression of tumor-associated trypsin inhibitor (TATI) and p53 in patients with bladder cancer undergoing radical cystectomy. Cancer Biomark 2019;26:281–9. 31594208 10.3233/CBM-182143 PMC12826419

[11] Nocito A Bubendorf L Tinner EM. Microarrays of bladder cancer tissue are highly representative of proliferation index and histological grade. J Pathol 2001;194:349–57. 11439368 10.1002/1096-9896(200107)194:3<349::AID-PATH887>3.0.CO;2-D

[12] Quintero A Alvarez-Kindelan J Luque RJ. Ki-67 MIB1 labelling index and the prognosis of primary TaT1 urothelial cell carcinoma of the bladder. J Clin Pathol 2006;59:83–8. 16394286 10.1136/jcp.2004.022939 PMC1860249

[13] Ziaran S Harsanyi S Bevizova K. Expression of E-cadherin, Ki-67, and p53 in urinary bladder cancer in relation to progression, survival, and recurrence. Eur J Histochem 2020;64:3098. 10.4081/ejh.2020.3098 PMC7118433 32214283

